# Patient Experiences Questionnaire for Out-of-Hours Care (PEQ-OHC) and EQ-5D-5L: reliability and validity following a national survey

**DOI:** 10.1080/02813432.2026.2757855

**Published:** 2026-11-03

**Authors:** Andrew M. Garratt, Kirsten Danielsen, Nina Berggaard, Steinar Hunskaar

**Affiliations:** ^a^ Division for Health Services, Norwegian Institute of Public Health, Oslo, Norway; ^b^ Department of Quality Indicators and User Surveys, Norwegian Directorate of Health, Oslo, Norway; ^c^ Department of Global Public Health and Primary Care, University of Bergen, Bergen, Norway; ^d^ National Centre for Emergency Primary Health Care, NORCE AS, Bergen, Norway

**Keywords:** EQ-5D-5L, PEQ-OHC, patient experiences, quality measurement, test-retest reliability, validity

## Abstract

**Background:**

The Patient Experiences Questionnaire for Out-of-Hours Care (PEQ-OHC) is used in local and national surveys of primary care but has not been evaluated for measurement properties since 2010. This study assessed aspects of validity, reliability, and internal consistency alongside the EQ-5D-5L.

**Methods:**

The PEQ-OHC and EQ-5D-5L were part of an electronic survey of 1,765 OHC patients recruited nationally across Norway 4–8 weeks following care. At 7–12 days, respondents received a retest questionnaire. Testing followed the COnsensus-based Standards for the selection of health Measurement INstruments (COSMIN) checklist for patient-reported instruments.

**Results:**

The 509 (29%) respondents had a median (IQR) age of 55 (38–66) years and 54% were female. Confirmatory factor analysis showed evidence for structural validity including PEQ-OHC scales of doctor contact, nurse contact, organisation and telephone contact (Comparative Fit/Tuckey Lewis Index = 0.97, Standardised Root Mean Residuals = 0.05). Together with a new scale of video contact, Cronbach’s alpha ranged from 0.79–0.98; only organisation fell below 0.90. Test-retest weighted kappa was generally of a high to excellent level for PEQ-OHC items (0.59–0.86) and EQ-5D dimensions (0.76–0.85). Intraclass correlation coefficients ranged from 0.74–0.83 for PEQ-OHC scale scores; EQ-5D-5L index = 0.91, EQ VAS = 0.81. Over 75% of results followed hypotheses, in comparisons of instrument scores with variables relating to experiences, health and services use.

**Conclusion:**

Evidence for reliability and validity further supports application of the PEQ-OHC in national and local quality measurement. The EQ-5D-5L also performed well and can be used alongside the PEQ-OHC to assess and adjust for case-mix across OHC centres.

## Introduction

Out-of-hours care (OHC) in Norway is a 24-hours public municipality led primary care service designed to ensure adequate acute care in emergency non-life-threatening situations [1]. It plays a central role in acute care and is widely used outside GP consultation times. There are 167 OHC centres in Norway and 16% of the population use the service annually [2,3].

Norwegian OHC services are obliged to take a systematic approach to quality improvement which includes the perceptions of service users, with a Patient Experiences Questionnaire (PEQ-OHC) specifically for this purpose [4,5]. The PEQ-OHC was developed for Norwegian settings following a systematic review of the content and measurement properties of patient-reported experience measures (PREMs) available for OHC [6]. Content was further informed by interviews with service users and consultation with an expert group in Norway, designed to ensure content validity of the PEQ-OHC as a measure of care quality from the patient perspective. It is brief with 15 items covering four aspects of care or scales of doctor contact, nurse contact, organisation, and telephone contact. Following a survey in 2008 and satisfactory evidence for structural validity, internal consistency and construct validity [5], the PEQ-OHC has been used in national and local surveys to assess the quality of out-of-hours acute care [4,5,7].

Since the PEQ-OHC was developed there have been important changes in the organisation of OHC services in Norway. First, there has been a policy shift towards telephone triage following the introduction of six-digit telephone number 116117, which patients are encouraged to call in advance [1]. There is variation in the use of triage but there has been a steady decrease in direct access to the service and increased use of telephone contact during this period [1,8,9]. Second, under the COVID-19 pandemic video consultation became available [10,11], a form of contact not covered by the PEQ-OHC with implications for content validity.

Changes in the organisation of OHC services in Norway have implications for the content validity and measurement properties of the PEQ-OHC which have not been tested since development 18 years ago. Moreover, the instrument has not been assessed for test-retest reliability, which not only contributes evidence for the stability of the PEQ-OHC scales, but also for single items widely used alongside PREMs including overall satisfaction with services [5,12]. The nature of such items precludes the more extensive testing of PREMs scale scores, including structural validity and internal consistency. Hence, evidence for test-retest reliability is necessary if such items are to be used as part of quality measurement. Test-retest can also inform standard error of measurement and smallest detectable change or difference of PEQ-OHC scales [13,14].

Since the PEQ-OHC was developed, the increasing use of patient-reported outcome measures (PROMs) alongside PREMs gives a more complete perspective of patient perceptions of healthcare quality and outcomes [15]. Crude single-item health measures have largely been used for case-mix adjustment when comparing different healthcare providers [16–18], such as OHC centres. Brief generic PROMs with satisfactory measurement properties serve a dual purpose of assessing variation in patient health and outcomes across centres, while enabling fair provider benchmarking.

The PEQ-OHC and EQ-5D-5L were included in a recent national survey of recipients of OHC. The PEQ-OHC has not undergone testing since 2010, and this is the first application of the EQ-5D-5L in this setting. The overall aim of the current study was to assess measurement properties in accordance with the COSMIN (COnsensus-based Standards for the selection of health Measurement INstruments) checklist [13,14], including whether the PEQ-OHC still has necessary evidence for use as a national quality indicator. The objectives were threefold. First, to develop a new PEQ-OHC scale of video contact. Second, to assess acceptability and content validity of the PEQ-OHC and video scale which included face-to-face interviews with patients. Third, following a cross-sectional national electronic survey, measurement properties relevant to both instruments were assessed including structural validity, internal consistency, test-retest reliability, and construct validity by means of hypothesis testing.

## Methods

### Development of the PEQ-OHC video contact scale

In 2025, an expert group was established and consulted on all aspects of the national survey including survey design and implementation, patient recruitment, and reporting. The group (*N* = 15) comprised OHC clinicians, OHC researchers, and representatives from patient organisations. The group reviewed the electronic version PEQ-OHC for content validity including the addition of the four-item video contact scale which was based on that for telephone contact. Free-text boxes were included for feedback, and further feedback was requested at a subsequent meeting of the expert group. Following any necessary changes to the questionnaire, cognitive interviews were then undertaken with 10 patients prior to the survey to assess acceptability and content validity of the PEQ-OHC including new video contact items. The interviews were undertaken with patients who had received care at Oslo OHC in June 2025 with recruitment designed to include patients of different ages, gender, and health problems. Patients were asked to complete the PEQ-OHC and were then asked about their understanding of items and content relevance.

### Cross-sectional survey

Following the interviews and any necessary changes to the PEQ-OHC, 1,765 patients who had received OHC were recruited to a cross-sectional national survey which included the PEQ-OHC, EQ-5D-5L and a retest component. The Norwegian Registry for Primary Health Care (NRPHC), which registers all consultations with municipal out-of-hours care in Norway [19], was used to select patients in August 2025 and 4–8 weeks following care. The registry undertakes continuous data collection, and this time frame also ensured data completeness following completion of the verification cycle. Patients were invited to participate through the national health portal for Norwegian residents, Helsenorge, a secure digital hub for personal health services. Following a request for them to log in using a secure-electronic ID, patients received a link to the survey information and questionnaire. The content was available in the two main Norwegian languages (Bokmål and Nynorsk) or English. Electronic reminders were sent to non-respondents at 6 and 20 days.

For purposes of assessing test-retest reliability, the final question asked if they would be willing to complete a shorter questionnaire 7–12 days later. This variation was due to administrative constraints but is still within the 1–2 weeks recommended by COSMIN [13,14]. To reduce respondent burden, patients who responded yes were randomized to either the PEQ-OHC or EQ-5D-5L.

The Data Protection Impact Assessment was approved by the Norwegian Directorate of Health on 6 May, 2025. The Health Data Service granted access to health data on 10 December, 2025 and exemption of duty of confidentiality on 21 August, 2025. Written informed consent was received from all respondents to the questionnaire. Patients were also given the opportunity to withdraw their questionnaire and/or opt out of the project’s collection of background information from the NRPHC and the Norwegian Patient Registry (NPR) [19] until 05 January 2026. None chose to withdraw or opt out.

### Patient-reported measures

The digital questionnaire began with questions relating the severity of the health problem and whether patient had tried to make an appointment with their GP before OHC was contacted. This was followed by the 19-item PEQ-OHC (Supplementary File 1) alongside single items relating to patient experiences, and EQ-5D-5L. The five PEQ-OHC scales each comprise four items except organisation, which has three. Scales scores are based on the sum of item responses where half or more items are completed, and are converted to 0 to 100 scale, where 100 is the best possible OHC experiences. Because items are equally weighted, the conversion follows a simple linear transformation of summed raw scores.

The single items are widely used in Norwegian surveys of patient experiences [5,12], and to minimize respondent burden while aiding recall, appear alongside PEQ-OHC scales if they assess related constructs. For example, items relating to waiting for examination/treatment appear alongside the PEQ-OHC scale items of organisation. Items assessing overall satisfaction and overall help arising from the OHC consultation come directly after the PEQ-OHC. All items have five-point descriptive scales and assess adequacy of information for the health problem, adequacy of information for the results of examinations/tests, overall satisfaction with the OHC service, overall help from the OHC service in relation to expectations, and whether they were incorrectly treated in any way by the OHC service (Supplementary File 1). Additional items specific to this survey included self-perceived severity of the problem/injury, waiting time for examination/treatment and whether they had received any follow-up since being in contact with the service (hospital admission, out-of-hours services, GP, outpatient clinic, other health professionals).

The EQ-5D-5L includes five items/dimensions (mobility, self-care, usual activities, pain/discomfort, anxiety/depression) with five response levels relating to ‘health today.’ Health states are transformed to a single index using a scoring algorithm derived from valuation tasks typically undertaken with general population samples to give a national value set [20]. Scores for the Norwegian EQ-5D-5L index range from −0.453 to 1; 1 is the best possible health state, and negative values represent states worse than dead, which is equal to 0 [20]. In addition, the EQ VAS assesses health on a vertical 0–100 visual analogue scale, with the endpoints of the worst imaginable and best imaginable health state. The EQ-5D-5L has undergone considerable testing in Norwegian populations and is the most widely used patient-reported measure in the national system of medical registers [20–22]. Permission for using the Norwegian EQ-5D-5L version 1 was granted by EuroQol (registration number 69998).

The follow-up retest questionnaire included either the PEQ-OHC together with the single items above, or the EQ-5D-5L. The latter included a transition item on whether general health had changed since completing the first questionnaire with a five-point scale of worse, slightly worse, about the same, slightly better, better.

### Register data

Questionnaire data were matched with several variables from NPR and NRPHC [19]: age, sex, ICPC2 main diagnosis, Charlson Comorbidity Index (CCI), use of mental health or substance use specialist health care services in the last two years (yes/no), and number of billings registered with either GP or OHC services in the last two years.

### Statistical analysis

Respondent characteristics are presented using number (%) and median (interquartile range) age, and the Charlson Comorbidity Index (CCI). Non-respondents and respondents to the survey were compared using first, the Wilcoxon rank sum test for age, CCI and health service use over the last two years (GP, out-of-hours care); second, Pearson’s chi-squared test for proportions of males and females and use of mental health or substance use specialist health care services over the last two years; and third, Fisher’s exact test with simulated p-value for ICPC-2 main diagnosis.

Measurement properties of the PEQ-OHC and EQ-5D-5L were assessed according to the COSMIN checklist [13,14]. Confirmatory factor analysis (CFA) was used to assess the structural validity of the PEQ-OHC, or the extent to which the item scores adequately contribute to scales [23]. Robust maximum likelihood (MLR) estimation was used because of the modest sample size and potential loss of statistical power through listwise deletion [24]. Model fit was assessed by the Comparative Fit Index (CFI), Tucker Lewis Index (TLI), Root Mean Square Error Approximation (RMSEA) and Standardised Root Mean Residuals (SRMR); acceptable if CFI/TLI > 0.95 or RMSEA < 0.06 or SRMR < 0.08 [13,25].

Internal consistency of the scales was assessed by Cronbach’s alpha where estimates of 0.70 and 0.90 are necessary for group and individual comparisons respectively [13,25], with the former being most relevant to national reporting at the OHC centre level [4,7,8].

Test-retest reliability of PEQ-OHC, EQ-5D-5L index and EQ VAS scores was assessed with the intraclass correlation coefficient (ICC) within a two-way mixed effects model with absolute agreement [13]. Kappa with quadratic weighting [26] was used for assessing individual item reliability, including single items used alongside the PEQ-OHC not contributing to scale scores. Kappa levels of ≥ 0.7 are deemed sufficient for single items [13]. The standard error of measurement (SEM) and smallest detectable change (SDC) were calculated. The former is the square root of the total error variance. For individuals the SDC is 1.96 × √2 × SEM and for groups, the SDC for individuals is divided by √*n* [13,25]. For the EQ-5D-5L, only patients who responded ‘about the same’ to the general health transition item were included in the test-retest analysis.

To assess construct validity in accordance with COSMIN guidelines, four hypotheses were formulated regarding the validity of the PEQ-OHC scale scores. First, PEQ-OHC scores were expected to have low-to-moderate correlations (0.2–0.5) with perceived ‘telephone waiting time,’ with the highest expected for the telephone contact scale. Second, scores were expected to have low-to-moderate correlations with both ‘waiting time for examination/treatment at the centre’ and ‘waiting room condition.’ These items relate to OHC functioning and general organisation, with highest correlations expected for the PEQ-OHC organisation scale. Third, low-to-moderate correlations (0.3–0.6) were expected between PEQ-OHC scales where clinicians play a primary role (doctor, nursing, and video contact) and perceptions of the ‘adequacy of information’ (regarding health problems and test results). Finally, consistent with previous validation studies [5], moderate-to-high correlations (0.4–0.7) were expected between PEQ-OHC scales and global assessment items, specifically overall satisfaction, overall help received, and perception of incorrect treatment.

EQ-5D-5L scores were compared to aspects of health and health care events in relation to the following hypotheses. First, musculoskeletal disorders, mental health problems, and cardiovascular disease make the largest contributions to years lived with disability in Norway [27,28] and hence, EQ-5D-5L index and EQ VAS scores would be poorer for patients with presenting diagnoses under these ICPC-2 classifications compared to the remainder of patients. For musculoskeletal disorders and cardiovascular disease, dimension scores would generally be poorer. For mental health, anxiety/depression dimension scores would be poorer. Second, EQ-5D-5L scores should reflect increasing levels of both the self-perceived severity of the presenting health problem and Charlson scores. Third, EQ-5D-5L index, EQ VAS and anxiety/depression dimensions scores would be poorer for those who had received mental health or substance use specialist health care services in the last two years. Fourth, poorer EQ-5D-5L scores would be associated with increases in health care billing relating to GP and OHC services in the last two years. Finally, compared to patients who had no follow-up, those who stated that they received follow-up after OHC were expected to have poorer EQ-5D-5L scores. Comparisons with binary variables used the Wilcoxon (dimensions) and t-test (index and EQ VAS). Comparisons with ordinal variables used the Jonckheere–Terpstra test for monotonic trend.

R version 4.5.1 with the Lavaan and Psych packages was used for statistical analyses.

## Results

### Content validity of the PEQ-OHC

The expert group agreed with the inclusion and content of the video contact scale and the revised PEQ-OHC was judged to assess the most important aspects of OHC experiences. Findings from the cognitive interviews with 10 patients further supported content validity and acceptability of the instrument. No changes were made to the PEQ-OHC.

### Cross-sectional survey

Table 1 shows the characteristics of the non-respondents and 509 (28.8%) respondents. Compared to non-respondents, respondents had a statistically significant older age (median difference 15 years), slightly higher Charlson scores, in the last two years they had 4% fewer consultations with specialist mental health/substance use services, and they had slightly higher use of GP services. Of those that responded to the first questionnaire, 225 (44.2%) were willing to complete the retest questionnaire comprising the PEQ-OHC or EQ-5D-5L, and 162 (72.0%) responded. The new video consultation scale could not be assessed for structural validity or reliability due to small sample sizes of 22 and 2, respectively. However, sample sizes were sufficient for preliminary testing for construct validity.

**Table 1.** Characteristics of non-respondents and respondents to the survey.

	Non-respondents *n* (%)	Respondents *n* (%)
	1,256	509 (29)
Age years median (IQR)^a^	40 (28–62)	55 (38–66)**
Female^b^	641 (51)	275 (54)
Male	615 (49)	234 (46)
Presenting diagnosis^c^		
General/unspecified^d^	339 (27)	125 (25)
Digestive	113 (9)	47 (9)
Cardiovascular	54 (4)	25 (5)
Skin/subcutaneous tissue	156 (12)	55 (11)
Airways	107 (9)	40 (8)
Musculoskeletal	175 (14)	93 (18)
Nervous system	75 (6)	26 (5)
Mental/behavioural	72 (6)	16 (3)
Genitourinary	56 (5)	32 (6)
Eye and adnexa	44 (4)	27 (5)
Other^e^	63 (6)	23 (4)
Charlson Comorbidity Index, median (IQR)^a^	0 (0–0)	0 (0–1)**
Mental health/substance use services past 2 years^b^		
No (%)	1,063 (85)	455 (89)
Yes (%)	193 (15)	54 (11)*
Health service use past 2 years^a^		
GP	4 (2–4)	4 (3–4)*
GP-OHC	1 (0–3)	1 (0–3)

^a^ Wilcoxon rank sum test.

^b^ Pearson’s chi-squared test.

^c^ Fisher’s exact test with simulated *p*-value used across all diagnoses. ICPC-2, International Classification of Primary Care.

^d^ General/unspecified refers to chapter A of the ICPC-2 classification system and comprises symptoms, conditions, and reasons for encounter that are general in nature, are not attributable to a specific organ system, or are unspecified with regard to their nature or location.

^e^ Other was used for presentational purposes and includes diagnoses comprising ≤ 10 respondents; testing was carried out on all diagnoses.

Asterisks denote statistical significance: *p < 0.05; **p < 0.01.

### Structural validity and internal consistency

CFA results showed good model fit for the four PEQ-OHC scales according to all four robust fit indices; CFI = 0.974, TLI = 0.966, RMSEA = 0.071, SRMR = 0.048. Supplementary Table 1 shows the factor loadings and correlations. Cronbach’s alpha ranged from 0.79 to 0.96, with only organisation falling below 0.90. The video consultation scale had an alpha of 0.98.

### Test-retest reliability

Table 2 shows that the percentage agreement for the test-retest reliability of PEQ-OHC items ranged from 58 to 75%, the majority (9/15) exceeding 65%. Quadratic weighted kappa ranged from 0.59 to 0.86, the majority (11/15) exceeding the criterion of 0.7. Items within the telephone contact scale had the lowest levels and kappa. ICCs ranged from 0.74 to 0.83 for telephone and doctor contact scales respectively (Table 2). Bubbleplots for the four test-retest scores are shown in Supplementary Figure 1. Being less dependent on the type of contact received, single items had slightly more respondents (Table 2). Those relating to information and overall help received had the lowest levels of percentage agreement and kappa; two did not meet the 0.7 criterion for kappa.

**Table 2.** Test-retest reliability for EQ-5D-5L, PEQ-OHC^a^ and single items.

Instrument scale/item	*n*	Agreement %	ICC/Kappa^b^
PEQ-OHC			
Doctor contact	47		0.83
Taken seriously		72.3	0.79
Interested in problem		74.5	0.86
Understandable		63.8	0.74
Competent		68.1	0.75
Nurse contact	40		0.77
Caring		57.5	0.70
Taken seriously		67.5	0.79
Understandable		65.0	0.71
Competent		60.0	0.67
Organisation	59		0.81
Information – waiting time		57.9	0.71
Waiting time acceptable		67.8	0.73
Centre well organised		63.8	0.77
Telephone contact	56		0.74
Taken seriously		69.6	0.64
Interested in problem		75.0	0.74
Understandable		73.2	0.59
Competent		64.3	0.62
Single items^c^	74		
Information – condition		51.4	0.71
Information – results		50.0	0.52
Overall satisfaction		67.6	0.80
Overall help		59.5	0.54
Incorrectly treated		79.7	0.72
EQ-5D-5L index	59		0.91
Mobility		76.3	0.76
Self-care		86.4	0.80
Usual activities		78.0	0.76
Pain/discomfort		59.3	0.81
Anxiety/depression		78.0	0.85
EQ VAS		—	0.81

^a^ The video contact scale was not included in the test-retest analysis due to insufficient data at retest (*n* = 2).

^b^ Intraclass correlation coefficient, quadratic weighted kappa. EQ-5D-5L reliability assessed for no change in general health transition at retest completion.

^c^ Do you think you were given enough information about your condition? Were you given enough information about the results of tests and investigations? Overall, was the help you received from the out-of-hours medical service satisfactory? Overall, how did you find the help you received from the out-of-hours medical service? Do you think you were treated incorrectly in any way by the out-of-hours medical service (as far as you are able to judge for yourself)?

PEQ-OHC test and retest scores were within 1–2 scale points on the 0–100 scale (Table 3). The SEM ranged from 7.71 to 11.19 for telephone contact and organisation scales respectively. The SDC for the comparison of groups ranged from 2.68 to 4.22 on the 0–100 scale.

**Table 3.** Q-5D-5L and PEQ-OHC^a^ standard error of measurement (SEM) and smallest detectable change (SDC).

	*n*	Test mean (SD)	Retest mean (SD)	SEM^b^	SDC^c^ individual	SDC^c^ group
PEQ-OHC^d^						
Doctor contact	47	84.97 (21.48)	83.24 (20.61)	8.68	24.06	3.51
Nurse contact	40	84.84 (21.55)	82.97 (19.20)	9.63	26.71	4.22
Organisation	59	66.81 (26.15)	65.61 (25.34)	11.19	31.02	4.04
Telephone contact	56	83.82 (15.49)	81.81 (14.66)	7.71	21.37	2.86
EQ-5D-5L index^e^	59	0.805 (0.266)	0.807 (0.255)	0.0784	0.217	0.028
Median (IQR)		0.928 (0.700–1)	0.876 (0.775–1)			
EQ VAS^f^	59	71.57 (21.62)	66.75 (21.16)	9.23	25.57	3.33
Median (IQR)		80 (57.5–89.5)	70 (50–81)			
Number (%) reporting problems						
Mobility	59	21 (35.6)	18 (30.5)			
Self-care	59	9 (15.3)	8 (13.6)			
Usual activities	59	22 (37.3)	21 (36.6)			
Pain/discomfort	59	40 (67.8)	41 (69.5)			
Anxiety/depression	59	20 (33.9)	23 (39.0)			

^a^ The video contact scale was not included in the test-retest analysis due to insufficient data at retest (*n* = 2).

^b^ Standard error of measurement.

^c^ Smallest detectable change.

^d^ PEQ-OHC scores range from 0 to 100 where 100 is the best possible experiences.

^e^ EQ-5D-5L index scores range from −0.45 to 1 where 1 is the best possible health state.

^f^ EQ VAS scores range from 0–100 where 100 is the best possible health state.

Of the 88 respondents to the EQ-5D-5L study arm, 59 (67%) responded that their general health was about the same at retest. For these respondents, EQ-5D-5L dimensions had percentage agreement ranging from 59% to 86% for pain/discomfort and self-care respectively (Table 2). Kappa ranged from 0.76 to 0.85; three of the dimensions were over 0.8. ICCs for the EQ-5D-5L index and EQ VAS were 0.91 and 0.81 respectively. Test- and retest scores were very similar for the index (Table 3). EQ VAS scores were approximately 5 points lower, and significantly different at retest, denoting poorer health. Bubbleplots for the index and EQ VAS are shown in Supplementary Figure 2. The SEM was 0.078 for the index and 9.23 for the EQ VAS. The SDC for groups was 0.028 for the index and 3.33 for the EQ VAS.

### Hypothesis testing for validity

Table 4 shows that correlations for the five PEQ-OHC scores were generally of a low to moderate level with those for single items. Video contact scale scores had a higher correlation than expected with ‘telephone waiting time’ and for all but the doctor/nurse contact scales, there were some correlations under 0.4 with the global items ‘overall help received’ and ‘perception of incorrect treatment.’ 25/32 (78%) of the correlations were within the magnitude hypothesised, meeting the COSMIN requirement of 75% [21]. Table 5 shows the EQ-5D-5L scores compared to health-related and health care use variables. 66/69 (96%) of the findings supported the hypotheses; anxiety/depression scores showed no association with self-perceived severity of the presenting problem and, self-care and anxiety/depression scores were not associated with referral to other health services.

**Table 4.** EQ-OHC correlations^a^ with aspects of health care process and global ratings (*n* = 428^b^).

	PEQ-OHC scales				
Item	Doctor contact	Nurse contact	Organisation	Telephone contact	Video contact
Waiting time – telephone^c^	0.31	0.35	0.36	0.43	0.60
Waiting time – examination/treatment	0.16	0.26	0.47	0.20	0.12 NS
Waiting room conditions	0.30	0.37	0.47	0.35	0.09 NS
Information – condition	0.60	0.51	0.41	0.41	0.55
Information – results	0.36	0.35	0.33	0.30	0.37 NS
Overall satisfaction	0.69	0.59	0.50	0.50	0.73
Overall help	0.44	0.39	0.37	0.33	0.41 NS
Incorrectly treated	0.48	0.41	0.32	0.37	0.37 NS

^a^ Kendall’s Tau correlations all statistically significant (*p* < 0.01) unless stated: NS, not significant.

^b^ Video contact (*n* = 22).

^c^ All items use five-point descriptive scales of ‘not at all’ to ‘to a very great extent’ unless stated. Did you think that it took a long time before you managed to make contact with the out-of-hours medical service by telephone? After you arrived at the out-of-hours medical service centre, how long did you have to wait before you went in for examination/treatment (six-point scale from <10 min to >3 h)? Did you find the waiting room satisfactory? Do you think you were given enough information about your condition? Were you given enough information about the results of tests and investigations? Overall, was the help you received from the out-of-hours medical service satisfactory? Overall, how did you find the help you received from the out-of-hours medical service (five-point scale of much worse than expected to much better than expected)? Do you think you were treated incorrectly in any way by the out-of-hours medical service (as far as you are able to judge for yourself)?

**Table 5.** EQ-5D-5L dimensions, EQ index and EQ VAS scores by ICPC-2 diagnosis, self-perceived severity, comorbidity and health care use (*N* = 509).

		% reporting problems^a^						
	*n*	Mobility	Self-care	Usual activities	Pain/discomfort	Anxiety/depression	EQ-5D-5L index mean (SD)	EQ VAS mean (SD)
All patients	509	30	16	37	61	31	0.833 (0.233)	72.08 (22.57)
Presenting diagnosis (ICPC-2)^b^								
Cardiovascular	25	48**	28*	60**	76	48*	0.773 (0.226)	62.88 (24.83)*
Mental health	16	19	13	31	69	75**	0.697 (0.266)*	57.00 (22.37)**
Muskuloskeletal	93	46**	28**	55**	80**	34	0.773 (0.261)**	69.20 (23.71)
Self-perceived severity^c^^,^^d^								
Did not know	47	—	—	—	—	—	—	—
Less severe	127	27	9	30	54	32	0.878 (0.175)	76.38 (22.48)
Severe	259	29	18	37	63	31	0.832 (0.230)	72.95 (21.58)
Very severe	73	40	22	49**	69*	29	0.782 (0.290)*	70.19 (21.05)**
Charlson comorbidity index^c^								
0	381	26	14	34	56	29	0.850 (0.223)	74.33 (22.32)
1	59	41	25	41	70	36	0.807 (0.232)	69.10 (20.45)
2	46	41	20	48	78	41	0.760 (0.280)	65.57 (21.72)
3 or more problems	23	52**	17**	52**	78**	35**	0.756 (0.255)**	55.39 (24.60)**
Mental health/substance use services past 2 years^b^								
No	455	30	16	36	60	27	0.844 (0.227)	73.21 (22.16)
Yes	54	35	19	46	67	68**	0.740 (0.264)**	62.52 (23.92)**
Health care events past 2 years^c^								
General practice								
0–4	54	15	4	15	30	7	0.961 (0.070)	87.06 (13.27)
5–9	65	22	9	31	48	22	0.891 (0.184)	77.63 (21.62)
10–14	69	16	15	28	52	15	0.899 (0.159)	78.78 (18.72)
15 or more	321	38**	20**	44**	71**	41**	0.785 (0.258)**	66.99 (23.07)**
Out-of-hours care								
0	203	20	12	26	46	23	0.897 (0.164)	77.65 (20.76)
1	108	37	19	38	65	28	0.833 (0.233)	73.10 (20.63)
2	65	26	12	37	66	29	0.837 (0.230)	71.03 (22.71)
3 or more	133	42**	21**	54**	77**	49**	0.733 (0.286)**	63.25 (24.06)**
Follow-up^b^								
No	44	27	14	34	59	25	0.873 (0.157)	77.32 (18.97)
GP	130	32	19	49	71	42	0.787 (0.272)*	68.22 (22.79)*
Other health services	22	36	9	50	72	18	0.867 (0.150)	70.09 (25.78)
Yes, hospital admission	97	41*	29*	55*	74*	30	0.766 (0.286)**	65.40 (24.65)**

^a^ Data shown as % problems for ease of presentation but tests relate to five-point dimension scores.

^b^ Binary variables were tested with the Wilcoxon (dimensions) and t-test (EQ-5D-5L index and EQ VAS).

^c^ Jonckheere–Terpstra (JT) test for monotonic trend.

^d^ How severe did you consider your medical problem/injury to be at the time the out-of-hours medical service was contacted?

Asterisks denote statistical significance: *p < 0.05; **p < 0.01.

## Discussion

This study followed widely accepted international standards [13,14,25] for testing structural validity, internal consistency, reliability and construct validity with satisfactory results. The inclusion of the PEQ-OHC video contact scale enhances instrument relevance to contemporary OHC services and following cognitive interviews with OHC patients, content validity was found to be satisfactory.

PROMs are increasingly used alongside PREMs and the EQ-5D-5L was found to perform satisfactorily in tests of reliability and hypothesis testing for validity. The EQ-5D-5L complements the PEQ-OHC by providing information on the health-related quality of life (HRQoL) of patients, including variation between OHC centres or other levels of measurement in quality indicators work. National PREMs surveys have traditionally used crude single item measures of health status for case-mix adjustment [16–18], whereas the EQ-5D-5L index has five items that assess important aspects of HRQoL with evidence for reliability and validity.

Testing reliability in accordance with the COSMIN checklist including test-retest reliability [13], was absent for 50% of 88 PREMs designed for a range of health care settings included in a systematic review [29]. Test-retest studies that meet COSMIN criteria can also inform understanding of measurement error and agreement, and yet the SEM and SDC were only assessed for 2 of the 88 PREMs included in the review [29]. The results of the current study show adequate levels of test-retest reliability for the PEQ-OHC and the inclusion of the SEM and SDC can inform reporting of PEQ-OHC scores as part of quality measurement and improvement. There was no evidence for systematic differences between test and retest scores. In group level comparisons that are based on OHC centres, the SEM helps distinguish true differences in quality from measurement instability. The SDC is the minimum level of change in PEQ-OHC scores needed for a quality improvement initiative to be judged as clinically effective. Moreover, the SDC can be used to distinguish between differences between OHC centres in cross-sectional surveys, including those at the national level. Such information will further inform resource allocation within healthcare decision making that includes PREMs data.

The EQ-5D-5L dimension and index scores also showed high levels of reliability which, with excellent levels of Kappa (0.75–1) as defined by a systematic review of measurement properties for the instrument, are among the highest across studies that met inclusion criteria [30]. The EQ VAS test-retest reliability estimate was also acceptable but there was evidence for systematic error with a lower mean score of just under 5 points at retest, denoting slightly poorer health albeit under levels denoted as being minimally important in previous studies [31]. Following a systematic review of international studies, it was concluded that this component of the EQ-5D shows inconsistent test-retest reliability although few studies had high-quality evidence [32]. The EQ VAS assesses health in general, and patients may include aspects of health not covered by the five EQ-5D-5L dimensions when responding to the former. Moreover, this aspect of the EQ-5D-5L is more cognitively challenging compared to the five dimensions which ask about specific aspects of health. Given the health care setting, it is possible that some OHC patients may have experienced changes in aspects of health following diagnosis and treatment that are not captured by the EQ-5D-5L dimensions. The low sample size prevented a subgroup analysis which might have identified the source of this systematic error.

For both instruments, the results of validity testing met the COSMIN criterion that greater than 75% of results met hypotheses. PEQ-OHC scores were compared with those for single items commonly used alongside PREMs including in the original testing [5,6]. Given that only a low number of respondents had video contact, it was reassuring to find that the new scale had evidence for construct validity, but further testing is recommended in larger samples, including structural validity alongside the other four scales. Interestingly, video contact scores had higher than expected correlations with telephone contact. This might be because telephone contact is used for assessing the need for and arranging video contact but more importantly, that the same health personnel are involved in both forms of contact. Given the limited testing for the new scale, these results are encouraging including the high level of correlation with overall satisfaction.

The results of the hypothesis testing for the EQ-5D-5L show that poor health associated with diagnoses and health care use are being assessed across the dimensions, index and EQ VAS scores. EQ-5D-5L scores distinguished between important groups including patients presenting with somatic and mental health problems, along with self-perceived severity of the presenting health problem at the OHC service. The lack of baseline measurement limits the usefulness of the EQ-5D-5L in this context as a measure of outcome that can supplement the PEQ-OHC which assesses processes rather than outcomes. However, compared to single item measures of general health it has greater reliability and validity for purposes of case-mix adjustment, and it gives a more detailed description of HRQoL that can inform decision making relating to resources across OHC centres.

### Study strengths and limitations

The survey included a test-retest component where adequate response rates are often a challenge. The response rate of 29% is among the lowest for recent surveys of primary care patients in Norway [33–35], with digital surveys found to give lower response rates [33]. Respondents had a median higher age of 15 years, were more likely to be female, had poorer health in general as assessed by the CCI and use of GP services, but less contact with mental health and substance use specialist health care services in the last two years. The inclusion of traditional modes of administration including pen and paper may have improved response rates, particularly among patients who are not digitally active and hence do not use Helsenorge, the national health portal for Norwegian residents. Response bias may not have affected the results of testing including test-retest reliability in the current study, but it has implications for the design and conduct of future surveys of OHC services for purposes of quality measurement and quality improvement. Strategies to enhance response rates and/or alternative methods to assess experiences of health care are necessary if surveys are to be inclusive and representative.

The 4 to 8-week period between the OHC consultation and when patients received the questionnaire link is another important limitation which may affect recall of patient experiences. The survey is dependent on adequate registry data and while the Norwegian Registry for Primary Health Care (NRPHC) undertakes continuous data collection, data completeness is subject to a verification cycle [19].

The main study limitation arises from the small sample size and particularly for the test-retest. Test-retest results were generally very good but the use of more specific transition criteria relating to individual EQ-5D-5L dimensions would have been possible with a larger sample size. Testing for structural validity by means of CFA informed the sample size. However, information on the proportion of video contacts was unavailable and the results showed that a much larger sample size would have been necessary to include this scale in CFA. Structural validity testing with the addition of the video contact items, might follow a larger national survey and/or pooling data collection from local surveys.

The test-retest component contributed important information for interpretation of PEQ-OHC scores, including the SEM and SDC, aspects of PREMs testing that are largely lacking from development and testing [29]. However, the assessment of minimal important change/differences in PEQ-OHC scores would have further contributed to scale score interpretation [13,14] for purposes of quality measurement and quality improvement [29]. Such information is available for the EQ-5D-5L, but a range of estimates exist based on studies of varying quality across different populations [31,36].

## Conclusion

Following continued application of the PEQ-OHC in local and national surveys since its development in 2008, the evidence found for measurement properties by the current study is timely and supports continued use as a quality indicator. Content validity was enhanced through the inclusion of a scale of video contact, and while evidence for internal consistency and validity was satisfactory further testing is recommended. The EQ-5D-5L had good evidence for reliability and validity in OHC centre patients and will contribute to case-mix adjustment in quality measurement.

## Supplementary Material

IPRI_2026_0150_SuppFile1.docx

## Data Availability

The data is under consideration for being made available in anonymised form. Access will require an application to the Norwegian Directorate of Health.
