# Supplementary material for: Patient Experiences Questionnaire for Out-of-Hours Care (PEQ-OHC) and EQ-5D-5L: reliability and validity following a national survey
Source: Scand J Prim Health Care. 2026 Nov 3;44(1):2757855. doi: 10.1080/02813432.2026.2757855 (PMC13757831; doi:10.1080/02813432.2026.2757855)
Supplement: Supplementary file 1 [file IPRI_A_2757855_SM1827.docx]

**Supplementary file 1**

**Patient Experiences Questionnaire for Out-of-Hours Care (PEQ-OHC), English version**

**Telephone contact**

**About the staff you spoke to over the telephone**

The questions below concern the person you spoke to over the telephone when you called the out-of-hours medical service.

**Did you find that the person you spoke to took you seriously?**

Not at all

To a small extent

To some extent

To a large extent

To a very large extent

**Did you find that the person you spoke to was interested in the description of your condition?**

Not at all

To a small extent

To some extent

To a large extent

To a very large extent

**Did you find that the person you spoke to, spoke to you in a way that you could understand him/her?**

Not at all

To a small extent

To some extent

To a large extent

To a very large extent

**Did you find that the person you spoke to was professionally competent?**

Not at all

To a small extent

To some extent

To a large extent

To a very large extent

**Video calls**

**About the staff you spoke to on the video call**

The questions below concern the person you spoke to on the video call when you called the out-of-hours medical service.

**Did you find that the person you spoke to took you seriously?**

Not at all

To a small extent

To some extent

To a large extent

To a very large extent

**Did you find that the person you spoke to was interested in the description of your condition?**

Not at all

To a small extent

To some extent

To a large extent

To a very large extent

**Did you find that the person you spoke to, spoke to you in a way that you could understand him/her?**

Not at all

To a small extent

To some extent

To a large extent

To a very large extent

**Did you find that the person you spoke to was professionally competent?**

Not at all

To a small extent

To some extent

To a large extent

To a very large extent

**Doctor contact**

**About the doctors at the out-of-hours medical service centre**

The questions below concern your meeting with the doctor(s) at the out-of-hours medical service centre.

**Did you find that the doctor(s) took you seriously?**

Not at all

To a small extent

To some extent

To a large extent

To a very large extent

**Did you find that the doctor(s) was interested in the description of your condition?**

Not at all

To a small extent

To some extent

To a large extent

To a very large extent

**Did you find that the doctor(s) spoke to you in a way that you could understand him/her?**

Not at all

To a small extent

To some extent

To a large extent

To a very large extent

**Did you find that the doctor(s) were professionally competent?**

Not at all

To a small extent

To some extent

To a large extent

To a very large extent

**Nurse contact**

**About the nurses the out-of-hours medical service centre**

The questions below concern your meeting with the nurse(s) at the out-of-hours medical service centre.

**Did you find that the nurse(s) cared about you?**

Not at all

To a small extent

To some extent

To a large extent

To a very large extent

**Did you find that the nurse(s) took you seriously?**

Not at all

To a small extent

To some extent

To a large extent

To a very large extent

**Did you find that the nurse(s) spoke to you in a way that you could understand him/her?**

Not at all

To a small extent

To some extent

To a large extent

To a very large extent

**Did you find that the nurse(s) was professionally competent?**

Not at all

To a small extent

To some extent

To a large extent

To a very large extent

**Organisation**

**Did you receive satisfactory information about how long you should expect to wait before you would go in for examination/treatment?**

Not at all

To a small extent

To some extent

To a large extent

To a very large extent

**Do you think the length of time you had to wait from arriving until you were examined/treated was acceptable?**

Not at all

To a small extent

To some extent

To a large extent

To a very large extent

**Did you get the impression that the out-of-hours medical service was well organised?**

Not at all

To a small extent

To some extent

To a large extent

To a very large extent

**Single items**

**Do you think you were given enough information about your condition?**

Not at all

To a small extent

To some extent

To a large extent

To a very large extent

**Were you given enough information about the results of tests and examinations?**

Not at all

To a small extent

To some extent

To a large extent

To a very large extent

*Not applicable*

**Overall, was the help you received from the out-of-hours medical service satisfactory?**

Not at all

To a small extent

To some extent

To a large extent

To a very large extent

**Overall, how did you find the help you received from the out-of-hours medical service?**

Much worse than expected

A little worse than expected

As expected

A little better than expected

Much better than expected

**Do you think you were treated incorrectly in any way by the out-of-hours medical service (as far as you are able to judge for yourself)?**

Not at all

To a small extent

To some extent

To a large extent

To a very large extent
